# Survival Outcomes of Breast-Conserving Therapy versus Mastectomy in Early-Stage Breast Cancer, Including Centrally Located Breast Cancer: A SEER-Based Study

**DOI:** 10.1155/2022/5325556

**Published:** 2022-08-27

**Authors:** Tianshui Yu, Weilun Cheng, Ting Wang, Ziang Chen, Yu Ding, Jianyuan Feng, Yunqiang Duan, Anbang Hu, Mingcui Li, Hanyu Zhang, Yanling Li, Fei Ma, Baoliang Guo

**Affiliations:** ^1^Department of General Surgery, The Second Affiliated Hospital of Harbin Medical University, Harbin 150081, China; ^2^Department of General Surgery, Daqing Oilfield General Hospital, Daqing 163316, China

## Abstract

**Purpose:**

This study aims to analyze the survival outcomes of breast cancer (BC) patients, especially centrally located breast cancer (CLBC) patients undergoing breast-conserving therapy (BCT) or mastectomy.

**Methods:**

Surveillance, epidemiology, and end results (SEER) data of patients with T1-T2 invasive ductal or lobular breast cancer receiving BCT or mastectomy were reviewed. We used X-tile software to convert continuous variables to categorical variables. Chi-square tests were utilized to compare baseline information. The multivariate logistic regression model was performed to evaluate the relationship between predictive variables and treatment choice. Survival outcomes were visualized by Kaplan–Meier curves and cumulative incidence function curves and compared using multivariate analyses, including the Cox proportional hazards model and competing risks model. Propensity score matching was performed to alleviate the effects of baseline differences on survival outcomes.

**Result:**

A total of 180,495 patients were enrolled in this study. The breast preservation rates fluctuated around 60% from 2000 to 2015. Clinical features including invasive ductal carcinoma (IDC), lower histologic grade, smaller tumor size, fewer lymph node metastases, positive ER and PR status, and chemotherapy use were independently correlated with BCT in both BC and CLBC cohorts. In all the classic Cox models and competing risks models, BCT was an independent favorable prognostic factor for BC, including CLBC patients in most subgroups. In addition, despite the low breast-conserving rate compared with tumors located in the other areas, CLBC did not impair the prognosis of BCT patients.

**Conclusion:**

BCT is optional and preferable for most early-stage BC, including CLBC patients.

## 1. Introduction

Breast-conserving therapy (BCT), which refers to breast-conserving surgery plus postoperative radiotherapy, is considered a standard treatment for early-stage breast cancer. Several clinical trials, including NSABP B-06, Milan, and EORTC 10801, have proven that the survival outcomes of patients treated with BCT are equivalent to those undergoing mastectomy, despite a relatively higher risk of local recurrence [1–3]. In addition, BCT patients had significantly improved body image, satisfaction with treatment and sexual functioning, and there was no significant difference in fear of recurrence between patients treated with BCT and mastectomy [4, 5].

Centrally located breast cancer (CLBC) usually refers to tumors located in the area within 2 cm of the nipple-areola complex (NAC) but without NAC involvement. Because of the particularity of its position, surgeons are often not inclined to perform BCT in CLBC. To date, there are only limited studies focused on the safety and prognosis of BCT compared with mastectomy in CLBC, and none of these studies are comprehensive enough [6–8].

To this end, we conducted a detailed retrospective study based on the SEER database to evaluate the prognosis of BC patients undergoing BCT and mastectomy, especially CLBC patients. Moreover, we used both the classic Cox proportional hazards model and competing risks model to ensure the rigor of this research and reduce statistical errors. Furthermore, we performed a series of subgroup analyses to help surgeons make the best choice according to the patient's baseline information.

## 2. Materials and Methods

### 2.1. Participants

The data for this study were extracted from research plus data from 18 registries of the Surveillance, Epidemiology, and End Results (SEER) database released in November 2020. We enrolled 180,495 female patients who received mastectomy or BCT (breast-conserving surgery plus postoperative radiotherapy) after being diagnosed with primary T1-T2 invasive ductal carcinoma (IDC) or invasive lobular carcinoma (ILC) between 2000 and 2015. Patients over 80 years old; with breast cancer located in the nipple-areolar complex or axillary tail; Tis or T1 mic; with more than one primary cancer; having metastasis at diagnosis; initially identified at death or autopsy only; with unknown information on essential parameters; or missing in follow-up were excluded from the study. Asian, Pacific Islander, American Indian, and Alaska native were regarded as other races. Borderline ER or PR status was considered unknown status. Informed consent was not required because personally identifiable information was not accessed. Institutional Review Board permission was not required because the SEER database is a deidentified national database.

### 2.2. Statistical Analysis

Demographic information and clinical characteristics were compared using Chi-square tests. Continuous variables were converted to categorical variables using *X*-tile software (Version 3.6.1) [9]. Multivariable logistic regression was utilized to evaluate the relationship between predictive variables and treatment choice. We used the Kaplan–Meier curve to estimate survival outcomes, and the log-rank test was used to perform between-group comparisons. The Cox proportional hazards model was performed to fit demographic and clinical characteristics for overall survival (OS) and breast cancer-specific survival (BCSS). A1 : 1 ratio propensity score matching (PSM) method with a caliper of 0.02 was performed to alleviate the influence of baseline differences on survival outcomes in CLBC and upper-outer breast cancer (UOBC) patients who underwent BCT. Matching variables included the year of diagnosis, age, race, histological type, laterality, T stage, N stage, ER status, PR status, and chemotherapy status. Since the Cox regression model might not accurately estimate the risk of a particular event when competing risks exist, we performed the competing risks analysis to better evaluate the relationship between therapeutic strategies and survival outcomes. We treated death from other causes as a competing event. The risk of death caused by breast cancer was estimated using the cumulative incidence function curve and compared across groups using Gray's test. The Fine–Gray model (also known as the subdistribution hazard function) and the cause-specific (CS) model were applied for multifactor competing risks analyses. A *p*-value < 0.05 was considered statistically significant. Statistical analyses were performed using SPSS software (version 26.0, IBM Corporation) and SAS software (version 9.4, SAS Institute).

## 3. Results

### 3.1. Baseline Characteristics and the Trend of Breast-Conserving Therapy (BCT) and Mastectomy among Breast Cancer (BC) Patients

**Figure 1:**
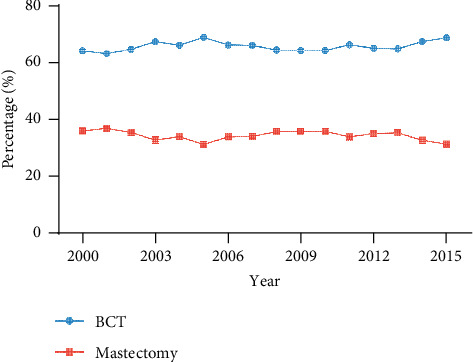
The proportion of BC patients undergoing BCT or mastectomy from 2000 to 2015. BCT, breast-conserving therapy.

According to our inclusion criteria, 180,495 patients were enrolled for analysis, among whom 118,552 (65.7%) patients received BCT and 63,963 (34.3%) patients underwent mastectomy. The clinical characteristics are displayed in Table 1. Patients between 64 and 72 years old, white patients, patients with the histology of invasive ductal carcinoma (IDC), and patients with less aggressive characteristics including histologic grades I and II, T1 stage, N0 stage, and positive ER and PR status were more inclined to receive BCT. In addition, patients who underwent BCT were less likely to receive chemotherapy.  Figure 1 shows the trend of BCT and mastectomy for the indicated patients from 2000 to 2015, and the breast preservation rates fluctuated around 60%.

### 3.2. Predictive Factors of BCT among BC Patients

Variables that were statistically significant (*p* < 0.05) in univariate analysis were enrolled in the multivariate logistic regression model. The multivariate analysis further validated that clinical features including diagnosis between 2004 and 2007, age between 64 and 72, black race, IDC, lower histologic grade, smaller tumor size, fewer lymph node metastases, positive ER and PR status, and chemotherapy use were independently correlated with BCT compared with mastectomy (Table 2).

### 3.3. Survival Analysis among BC Patients Treated with BCT or Mastectomy and Subgroup Analysis

**Figure 2:**
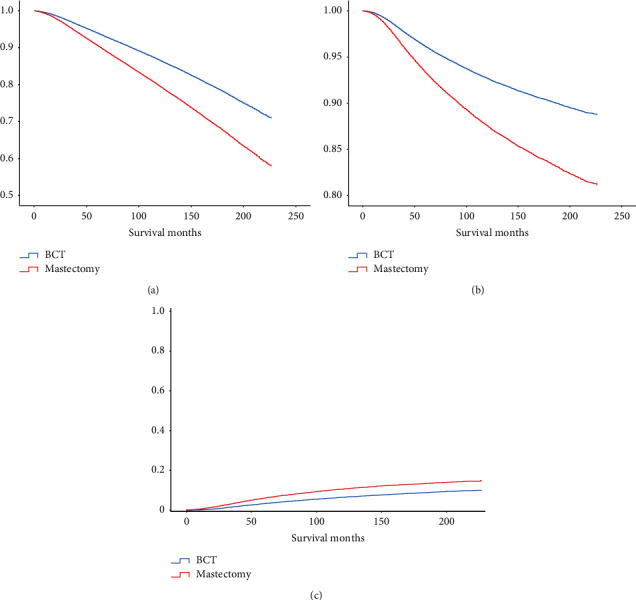
Survival outcomes of BC patients undergoing BCT or mastectomy. (a) Kaplan–Meier curve of OS. (b) Kaplan–Meier curve of BCSS. (c) Cumulative incidence function curve. BCT, breast-conserving therapy; OS, overall survival; BCSS, breast cancer-specific survival.

**Figure 3:**
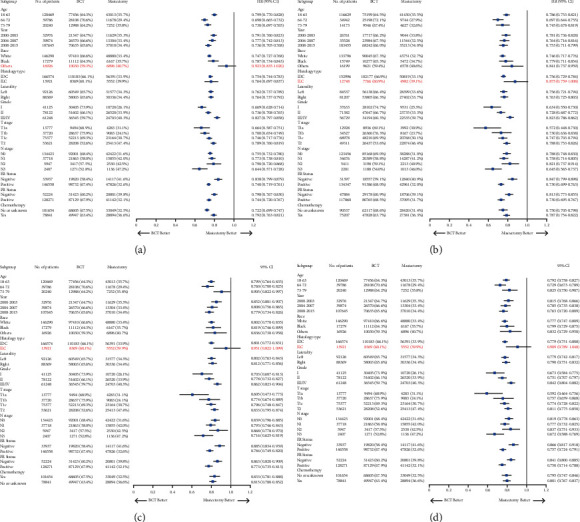
Survival outcome in each subgroup among BC patients. (a) OS in the Cox proportional hazards model. (b) BCSS in the Cox proportional hazards model. (c) Fine–Gray model in the competing risks analysis. (d) CS model in the competing risks analysis. OS, overall survival; BCSS, breast cancer-specific survival; CS, cause specific.

The Kaplan–Meier survival curve revealed that patients who received BCT had better overall survival (OS, *p* < 0.001) and breast cancer-specific survival (BCSS, *p* < 0.001) than those who underwent mastectomy (Figures 2(a) and 2(b)). The cumulative incidence function curve also showed that patients undergoing BCT had a lower risk of breast cancer-associated death (Figure 2(c)). Then, we conducted the Cox proportional hazards model and the competing risks models for the multivariate analyses (Table 3). The results obtained from the Cox model indicated that the independent risk factors associated with the OS and BCSS of BC patients included the year of diagnosis, age, race, histological type, histologic grade, *T* stage, N stage, ER status, PR status, and chemotherapy status. Notably, BCT was found to be a favorable prognostic factor for OS (HR 0.764, 95% CI 0.745–0.783, *p* < 0.001) and BCSS (HR 0.760, 95% CI 0.734–0.787, *p* < 0.001). Similar results were obtained from competing risks models. BCT was still an independent risk factor in the Fine–Gray model (HR 0.807, 95% CI 0.779–0.837, *p* < 0.001) and the CS model (HR 0.784, 95% CI 0.757–0.812, *p* < 0.001). The subgroup analysis further demonstrated that patients treated with BCT had significantly better prognoses than those who received mastectomy in nearly all subgroups, except for patients of other races in the OS model and ILC patients in the BCSS, Fine–Gray, and CS models (Figure 3).

### 3.4. Differences in Breast Preservation Rate among BC Patients with Distinct Tumor Locations

**Figure 4:**
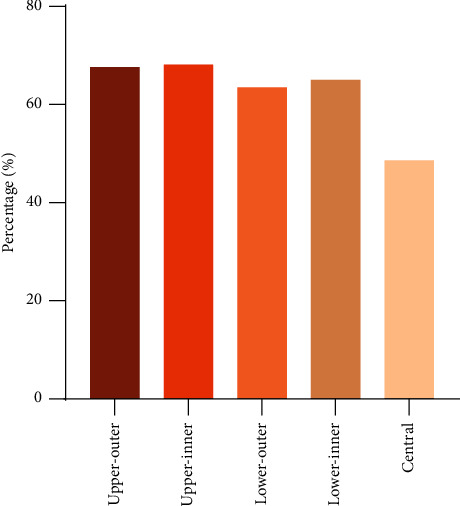
The proportion of BC patients with different tumor locations undergoing BCT or mastectomy between 2000 and 2015. BCT, breast-conserving therapy.

To detect the breast preservation rate of BC patients with different primary tumor locations, we divided the whole cohort into five subgroups: centrally located breast cancer (CLBC, *n* = 12,051), upper-outer breast cancer (UOBC, *n* = 97,517), upper-inner breast cancer (UIBC, *n* = 34,752), lower-outer breast cancer (LOBC, *n* = 20,091), and lower-inner breast cancer (LIBC, *n* = 16,084). Strikingly, except for CLBC group patients, more than 60% of patients received BCT in the other four subgroups. In the CLBC group, the breast-conserving rate of patients was only 48.7% (Figure 4).

### 3.5. Predictive Factors of BCT among CLBC Patients

The clinical characteristics of CLBC patients are shown in Table 4. Patients between 64 and 72 years old, patients of the white race, patients with IDC, and patients with less aggressive characteristics including histologic grades I and II, T1 stage, N0 stage, ER positivity, and PR positivity tended to receive BCT. Similarly, chemotherapy was less likely to be used for patients who underwent BCT.

In the multivariate logistic regression model, features including age between 64 and 72, white race, IDC, histologic grades I and II, T1 stage, N0 stage, positive PR status, and chemotherapy use were independently associated with BCT compared with mastectomy (Table 5).

### 3.6. Survival Analysis among CLBC Patients Treated with BCT and Mastectomy

**Figure 5:**
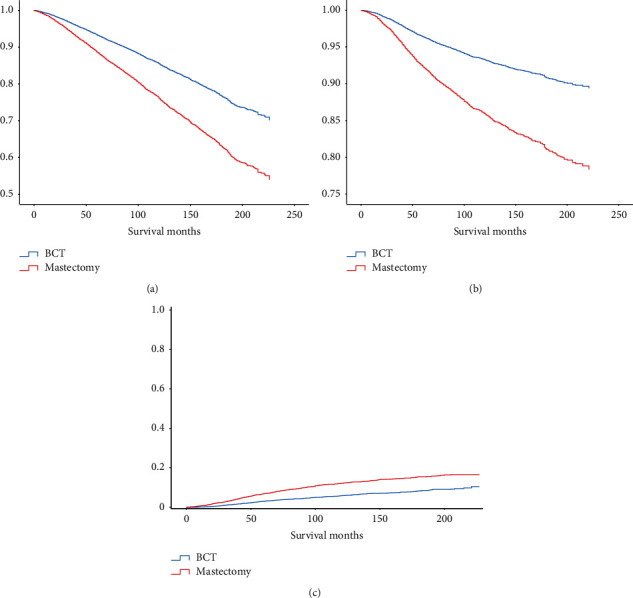
Survival outcomes of CLBC patients undergoing BCT or mastectomy. (a) Kaplan–Meier curve of OS. (b) Kaplan–Meier curve of BCSS. (c) Cumulative incidence function curve. CLBC, centrally located breast cancer; BCT, breast-conserving therapy; OS, overall survival; BCSS, breast cancer-specific survival.

The Kaplan–Meier survival curve showed that CLBC patients treated with BCT had enhanced overall survival (OS, *p* < 0.001) and breast cancer-specific survival (BCSS, *p* < 0.001) compared with those who underwent mastectomy (Figures 5(a) and 5(b)). Besides, the cumulative incidence function curve showed that CLBC patients who received BCT were less likely to die from breast cancer (Figure 5(c)). Moreover, the Cox proportional hazards model indicated that the year of diagnosis, age, race, histologic grade, T stage, N stage, ER status, PR status, and chemotherapy status were independent risk factors associated with the OS and BCSS of CLBC patients. BCT was also found to be a favorable prognostic factor for OS (HR 0.734, 95% CI 0.672–0.802, *p* < 0.001) and BCSS (HR 0.660, 95% CI 0.576–0.755, *p* < 0.001).

In competing risks analyses, BCT was still an independent favorable prognostic factor in the Fine–Gray model (HR 0.709, 95% CI 0.617–0.815, *p* < 0.001) and the CS model (HR 0.686, 95% CI 0.598–0.786, *p* < 0.001). However, the black race, which was proven to be a risk factor in the Cox model, was nonsignificant in the Fine–Gray model (*p*=0.133) and the CS model (*p*=0.109) (Table 6).

**Figure 6:**
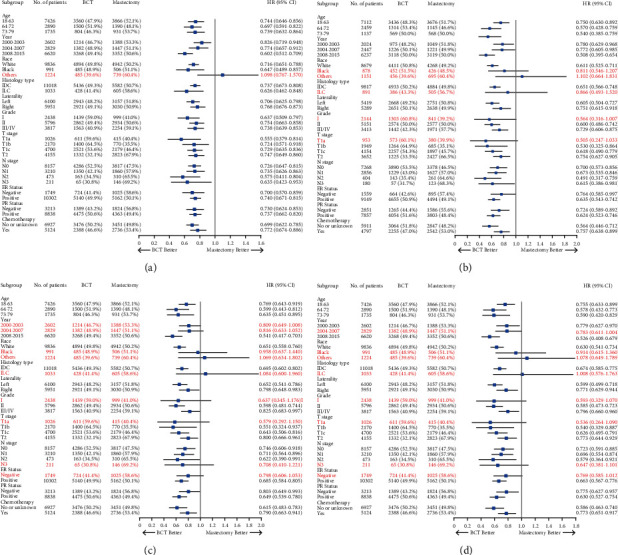
Survival outcome in each subgroup among CLBC patients. (a) OS in the Cox proportional hazards model. (b) BCSS in the Cox proportional hazards model. (c) Fine–Gray model in the competing risks analysis. (d) CS model in the competing risks analysis. CLBC, centrally located breast cancer; OS, overall survival; BCSS, breast cancer-specific survival; CS, cause specific.

The subgroup analysis indicated that patients treated with BCT had significantly better OS in almost all subgroups, except for patients of other races. Furthermore, patients who received BCT shared improved BCSS except for patients of black or other races, with ILC, histologic grade I, and T1a stage compared with those who underwent mastectomy. In the competing risks analyses, BCT patients had better prognoses except for those diagnosed between 2000 and 2007, of black or other races, with ILC, histologic grade I, T1a stage, N3 stage, and negative ER status (Figure 6).

### 3.7. Survival Analysis of BCT Patients with Differentially Located Tumors

To further reveal the safety and prognosis of BCT in CLBC patients, we performed survival analyses among patients with tumors located in five distinct areas (Table 7). When compared to UOBC, despite a worse OS (HR 0.932, 95% CI 0.869–0.999, *p*=0.047) in CLBC, tumors located in these two areas shared a similar BCSS (*p*=0.319) and had no significant difference in the Fine–Gray model (*p*=0.578) and the CS model (*p*=0.482) (Table 7, Table S1). Then, due to the huge differences in the patient number and clinical characteristics, we conducted propensity score matching (PSM) to reduce the influence of confounding factors. After matching, 5,864 patients in each cohort were enrolled. The results showed that patients with CLBC and UOBC had comparable prognoses in all models except the Cox-OS model (Table 7, Table S2). Intriguingly, CLBC patients showed improved prognoses when compared to those with UIBC (Table 7, Table S3), LOBC (Table 7, Table S4), and LIBC (Table 7, Table S5). Subsequently, we performed subgroup analyses among patients with CLBC and those with UIBC (Figure S1), LOBC (Figure S2), and LIBC (Figure S3). Patients in CLBC group showed similar prognoses compared to those with UIBC and LOBC in nearly all subgroups. When compared to LIBC, CLBC patients had better prognoses in most subgroups.

In addition, to explore whether the difference in prognosis between CLBC and LIBC is caused by internal mammary node (IMN) metastasis, we performed a survival analysis among patients without IMN metastasis in these two cohorts. The results indicated that compared to LIBC, CLBC was still an independent favorable prognostic factor among BCT patients (Table S6).

## 4. Discussion

To the best of our knowledge, this is the first population-based retrospective study using the competing risks model to evaluate the prognosis of TI-T2 CLBC patients undergoing BCT or mastectomy.

Several clinical trials have shown that patients treated with BCT and mastectomy have equivalent prognoses. For example, the NSABP B-06 trial demonstrated no significant differences in disease-free survival, distant-disease-free survival, or overall survival between early-stage BC patients treated with BCT and mastectomy [1]. The DBCG-82TM trial also showed no significant difference in 10-year recurrence-free survival and 20-year overall survival between these two groups [10]. Several studies even showed improved BCSS and OS for BCT compared with mastectomy [11, 12]. Moreover, BCT also achieved superior cosmetic outcomes than mastectomy [4]. However, our research demonstrated that nearly 40% of early-stage BC patients received mastectomy each year between 2000 and 2015, and this proportion increased to over 50% in CLBC patients. The hesitation of surgeons performing BCT for CLBC patients may be partially due to the special location or anatomic structure of tumors, including the complex lymphatic drainage [13]. Although a recent retrospective study based on the SEER database discussed the benefit of BCT in CLBC patients, only the classic Cox proportional hazards model was applied, and detailed subgroup analysis was absent [14]. Zhang's study compared the prognosis of breast-conserving surgery and mastectomy, but the postoperative radiotherapy status was not controlled [8].

In this study, we revealed a higher proportion of IDC, lower histologic grade, T stage and N stage, and positive ER and PR status to receive BCT for BC, including CLBC patients. These factors were mostly associated with a smaller region or less malignant tumor. However, as the SEER database does not collect information on the sequence of chemotherapy and surgery, we could not clarify the influence of neoadjuvant chemotherapy on the choice of BCT or mastectomy. In addition, the status of endocrine therapy and Ki-67, which influence the survival of BC patients, was also unattainable from SEER [15, 16].

Our research demonstrated significantly improved OS and BCSS for BCT in both the whole BC cohort and CLBC alone cohort, which was concordant with previous studies [14, 17, 18]. Importantly, we performed the competing risks models, which take into account not only deaths caused by BC but also deaths caused by other events as well as their effects. We presented the outcomes of two competing risks models: the Fine–Gray model, which is appropriate for evaluating prognostic factors [19], and the CS model, which is more suitable for etiological research [20]. In line with the Cox model, both competing risks models showed better prognoses for BCT, whether in the entire BC cohort or CLBC cohort. These results further proved the safety and efficacy of BCT in the selected population. In addition, patients with ILC showed better survival outcomes than IDC patients, which aligned with earlier studies [21, 22]. When deeply dug, most subgroups of BC could benefit from BCT. All subgroup patients of CLBC showed at least equivalent prognoses receiving BCT compared with mastectomy, and some subgroups such as white race, IDC, lower N stage, and positive ER status could benefit from BCT. Combined with previous studies, patients with these beneficial factors could be more inclined to choose BCT in future clinical decisions [23].

When comparing survival outcomes of BCT in CLBC and other areas, we found that CLBC was comparable with UOBC in the Cox-BCSS model, Fine–Gray model, and CS model after propensity score matching and better than tumors located in the other three quadrants. Some studies have shown that LIBC is an unfavorable prognostic factor for early-stage BC patients, probably due to the higher possibility of IMN metastasis [24, 25]. However, our study showed that CLBC still had a better prognosis than LIBC among patients without IMN metastasis in the BCT cohort.

There are still some limitations in our research. First, we could not evaluate the influence of neoadjuvant chemotherapy on surgical choice and survival outcome. Information on local recurrence rates was also unavailable. Thus, we could not compare local recurrence rates as a secondary outcome between the BCT and mastectomy cohorts. In addition, we could not obtain data about the cosmetic results and satisfaction with body image after BCT. Finally, the status of endocrine therapy, Ki-67, and patients' income level was absent, which may introduce bias into our results.

In conclusion, utilizing the classic Cox proportional hazards model and competing risks model, our research not only revealed the superiority of BCT compared with mastectomy in most early-stage breast cancer but also proved that patients with CLBC could also obtain better prognoses from BCT.

## Figures and Tables

**Table 1:** Comparison of baseline characteristics between BC patients undergoing BCT and mastectomy from 2000 to 2015.

	Mastectomy		BCT		*p*-value
	*N*	%	*N*	%	
Year					< 0.001
2000–2003	11629	18.8%	21347	18.0%	
2004–2007	13304	21.5%	26570	22.4%	
2008–2015	37010	59.7%	70635	59.6%	

Age, years					< 0.001
18–63	43013	69.4%	77456	65.3%	
64–72	11678	18.9%	28108	23.7%	
73–79	7252	11.7%	12988	11.0%	

Race					< 0.001
White	48880	78.9%	97410	82.2%	
Black	6167	10.0%	11112	9.4%	
Others	6896	11.1%	10030	8.5%	

Histologic type					< 0.001
IDC	56391	91.0%	110183	92.9%	
ILC	5552	9.0%	8369	7.1%	

Laterality					0.698
Left	31577	51.0%	60549	51.1%	
Right	30366	49.0%	58003	48.9%	

Grade					< 0.001
I	10720	17.3%	30405	25.6%	
II	26520	42.8%	51602	43.5%	
III/IV	24703	39.9%	36545	30.8%	

T stage					< 0.001
T1a	4283	6.9%	9494	8.0%	
T1b	9083	14.7%	28637	24.2%	
T1c	23164	37.4%	52213	44.0%	
T2	25413	41.0%	28208	23.8%	

N stage					< 0.001
N0	42422	68.5%	92001	77.6%	
N1	15855	25.6%	21863	18.4%	
N2	2530	4.1%	3417	2.9%	
N3	1136	1.8%	1271	1.1%	

ER					< 0.001
Negative	14117	22.8%	19820	16.7%	
Positive	47826	77.2%	98732	83.3%	

PR					< 0.001
Negative	20801	33.6%	31423	26.5%	
Positive	41142	66.4%	87129	73.5%	

Chemotherapy					< 0.001
No or unknown	33049	53.4%	68605	57.9%	
Yes	28894	46.6%	49947	42.1%	

BCT, breast-conserving therapy; IDC, invasive ductal carcinoma; ILC, invasive lobular carcinoma; ER, estrogen receptor; PR, progesterone receptor.

**Table 2:** Multivariate logistic regression analysis of factors associated with BCT.

	OR	95% CI	*p*-value
Year			< 0.001
2000–2003 vs. 2008–2015	0.994	0.967–1.020	0.634
2004–2007 vs. 2008–2015	1.082	1.055–1.110	<0.001

Age, years			<0.001
64–72 vs. 18–63	1.306	1.273–1.340	<0.001
73–79 vs. 18–63	1.028	0.994–1.062	0.105

Race			< 0.001
Black vs. white	1.054	1.019–1.091	0.003
Others vs. white	0.747	0.722–0.772	<0.001

Histological type			
IDC vs. ILC	1.367	1.317–1.418	<0.001

Grade			< 0.001
I vs. III/IV	1.486	1.438–1.536	<0.001
II vs. III/IV	1.137	1.109–1.166	<0.001

T stage			<0.001
T1a vs. T2	1.805	1.729–1.884	<0.001
T1b vs. T2	2.487	2.409–2.568	<0.001
T1c vs. T2	1.869	1.825–1.915	< 0.001

N stage			<0.001
N0 vs. N3	1.413	1.300–1.537	<0.001
N1 vs. N3	0.985	0.905–1.072	0.725
N2 vs. N3	1.109	1.007–1.223	0.036

ER			
Negative vs. positive	0.874	0.843–0.906	<0.001

PR			
Negative vs. positive	0.844	0.819–0.870	<0.001

Chemotherapy			
No or unknown vs. Yes	0.701	0.684–0.719	<0.001

BCT, breast-conserving therapy; OR, odds ratio; CI, confidence interval; IDC, invasive ductal carcinoma; ILC, invasive lobular carcinoma; ER, estrogen receptor; PR, progesterone receptor.

**Table 3:** Multivariate survival analysis of prognostic factors among BC patients.

	Cox-OS			Cox-BCSS			Fine–Gray			CS		
	HR	95% CI	*p*-value	HR	95% CI	*p*-value	HR	—	*p*-value	HR	—	*p*-value
Year of diagnosis			< 0.001			<0.001						
2000–2003 vs. 2008–2015	1.427	1.382–1.472	<0.001	1.914	1.832–1.999	<0.001	1.607	1.539–1.678	<0.001	1.557	1.491–1.625	<0.001
2004–2007 vs. 2008–2015	1.196	1.158–1.234	<0.001	1.329	1.272–1.389	<0.001	1.226	1.173–1.282	<0.001	1.202	1.150–1.258	<0.001

Age, years			<0.001			<0.001						
64–72 vs. 18–63	2.388	2.318–2.459	<0.001	1.595	1.525–1.667	<0.001	1.314	1.256–1.374	<0.001	1.394	1.333–1.457	<0.001
73–79 vs. 18–63	4.891	4.744–5.043	<0.001	2.877	2.730–3.033	<0.001	1.662	1.574–1.755	<0.001	1.955	1.855–2.061	<0.001

Race			<0.001			<0.001						
Black vs. white	1.378	1.329–1.429	<0.001	1.343	1.279–1.411	<0.001	1.291	1.227–1.359	<0.001	1.318	1.255–1.384	<0.001
Others vs. white	0.711	0.677–0.748	<0.001	0.762	0.712–0.815	<0.001	0.800	0.747–0.856	<0.001	0.788	0.737–0.843	<0.001

Histology type												
ILC vs. IDC	0.906	0.864–0.949	<0.001	—	—	0.844	—	—	0.278	—	—	0.474

Laterality												
Right vs. Left	0.967	0.944–0.990	0.005	0.946	0.915–0.979	0.002	0.947	0.915–0.981	0.002	0.946	0.914–0.979	0.001

Grade			<0.001			<0.001						
II vs. I	1.180	1.139–1.223	<0.001	1.958	1.820–2.107	<0.001	1.943	1.807–2.089	<0.001	1.951	1.813–2.100	<0.001
III/IV vs. I	1.430	1.374–1.488	<0.001	2.719	2.521–2.933	<0.001	2.676	2.480–2.888	<0.001	2.703	2.505–2.916	<0.001

T stage			<0.001			<0.001						
T1b vs. T1a	1.240	1.161–1.325	<0.001	1.225	1.070–1.402	0.003	1.175	1.027–1.344	0.019	1.191	1.041–1,363	0.011
T1c vs. T1a	1.565	1.471–1.666	<0.001	2.177	1.924–2.464	<0.001	2.028	1.792–2.294	<0.001	2.084	1.842–2.359	<0.001
T2 vs. T1a	2.306	2.163–2.458	<0.001	3.779	3.339–4.278	<0.001	3.470	3.065–3.929	<0.001	3.615	3.193–4.092	<0.001

N stage			<0.001			<0.001						
N1 vs. N0	1.496	1.453–1.540	<0.001	1.958	1.880–2.038	<0.001	1.923	1.845–2.004	<0.001	1.948	1.870–2.028	<0.001
N2 vs. N0	2.617	2.494–2.746	<0.001	3.742	3.529–3.968	<0.001	3.565	3.352–3.792	< 0.001	3.705	3.494–3.929	<0.001
N3 vs. N0	4.340	4.084–4.611	<0.001	6.276	5.854–6.729	<0.001	5.985	5.546–6.459	<0.001	6.306	5.882–6.760	<0.001

ER												
Positive vs. negative	0.832	0.800–0.865	<0.001	0.789	0.749–0.832	<0.001	0.792	0.749–0.838	<0.001	0.790	0.749–0.833	<0.001

PR												
Positive vs. negative	0.834	0.806–0.862	<0.001	0.716	0.681–0.752	<0.001	0.723	0.687–0.761	<0.001	0.717	0.683–0.754	<0.001

Treatment												
BCT vs. mastectomy	0.764	0.745–0.783	<0.001	0.760	0.734–0.787	<0.001	0.807	0.779–0.837	<0.001	0.784	0.757–0.812	<0.001

Chemotherapy												
Yes vs. no or unknown	0.779	0.757–0.803	<0.001	0.955	0.916–0.996	0.032	–	–	0.063	–	–	0.928

HR, hazard ratio; CI, confidence interval; ILC, invasive lobular carcinoma; IDC, invasive ductal carcinoma; ER, estrogen receptor; PR, progesterone receptor; BCT, breast-conserving therapy.

**Table 4:** Comparison of baseline characteristics between CLBC patients undergoing BCT and mastectomy from 2000 to 2015.

	Mastectomy		BCT		*p*-value
	N	%	N	%	
Year					0.063
2000–2003	1388	22.4%	1214	20.7%	
2004–2007	1447	23.4%	1382	23.6%	
2008–2015	3352	54.2%	3268	55.7%	

Age, years					<0.001
18–63	3866	62.5%	3560	60.7%	
64–72	1390	22.5%	1500	25.6%	
73–79	931	15.0%	804	13.7%	

Race					<0.001
White	4942	79.9%	4894	83.5%	
Black	506	8.2%	485	8.3%	
Others	739	11.9%	485	8.3%	

Histological type					<0.001
IDC	5582	90.2%	5436	92.7%	
ILC	605	9.8%	428	7.3%	

Laterality					0.357
Left	3157	51.0%	2943	50.2%	
Right	3030	49.0%	2921	49.8%	
Grade					
I	999	16.1%	1439	24.5%	
II	2934	47.4%	2862	48.8%	
III/IV	2254	36.4%	1563	26.7%	

T stage					<0.001
T1a	415	6.7%	611	10.4%	
T1b	770	12.4%	1400	23.9%	
T1c	2179	35.2%	2521	43.0%	
T2	2823	45.6%	1332	22.7%	

N stage					<0.001
N0	3871	62.6%	4286	73.1%	
N1	1860	30.1%	1350	23.0%	
N2	310	5.0%	163	27.8%	
N3	146	2.4%	65	1.1%	

ER					<0.001
Negative	1025	16.6%	724	12.3%	
Positive	5162	83.4%	5140	87.7%	

PR					<0.001
Negative	1824	29.5%	1389	23.7%	
Positive	4363	70.5%	4475	76.3%	

Chemotherapy					<0.001
No or unknown	3451	55.8%	3476	59.3%	
Yes	2736	44.2%	2388	40.7%	

CLBC, centrally located breast cancer; BCT, breast-conserving therapy; IDC, invasive ductal carcinoma; ILC, invasive lobular carcinoma; ER, estrogen receptor; PR, progesterone receptor.

**Table 5:** Multivariate logistic regression analysis of factors associated with BCT among CLBC patients.

	OR	95% CI	*p*-value
Age, years			0.001
64–72 vs. 18–63	1.186	1.082–1.300	< 0.001
73–79 vs. 18–63	1.037	0.925–1.162	0.538

Race			< 0.001
Black vs. white	1.088	0.948–1.248	0.229
Others vs. white	0.663	0.584–0.752	< 0.001

Histological type			
IDC vs. ILC	1.361	1.187–1.560	< 0.001

Grade			< 0.001
I vs. III/IV	1.520	1.352–1.710	< 0.001
II vs. III/IV	1.193	1088-1.308	< 0.001

T stage			< 0.001
T1a vs. T2	2.884	2.479–3.354	< 0.001
T1b vs. T2	3.448	3.063–3.880	< 0.001
T1c vs. T2	2.271	2.075–2.486	<0.001

N stage			<0.001
N0 vs. N3	1.628	1.197–2.215	0.002
N1 vs. N3	1.203	0.882–1.640	0.243
N2 vs. N3	1.103	0.771–1.578	0.592

ER			
Negative vs. positive	0,881	0,765–1.014	0.078

PR			
Negative vs. positive	0.807	0.737–0.883	<0.001

Chemotherapy			
No or unknown vs. yes	0.692	0.632–0.758	<0.001

CLBC, centrally located breast cancer; BCT, breast-conserving therapy; OR, odds ratio; CI, confidence interval; IDC, invasive ductal carcinoma; ILC, invasive lobular carcinoma; ER, estrogen receptor; PR, progesterone receptor.

**Table 6:** Multivariate survival analysis of prognostic factors among CLBC patients.

	Cox-OS			Cox-BCSS			Fine–Gray			CS		
	HR	95% CI	*p*-value	HR	95% CI	*p*-value	HR	95% CI	*p -*value	HR	95% CI	*p*-value
Year of diagnosis			<0.001			<0.001						
2000–2003 vs. 2008–2015	1.329	1.194–1.479	<0.001	2.005	1.715–2.344	<0.001	1.629	1.395–1.903	< 0.001	1.536	1.315–1.795	<0.001
2004–2007 vs. 2008–2015	1.134	1.016–1.265	0.025	1.441	1.226–1.693	<0.001	1.310	1.114–1.541	0.001	1.267	1.078–1.488	0.004

Age, years			< 0.001			<0.001						
64–72 vs. 18–63	2.437	2.200–2.700	<0.001	1.757	1.514–2.039	<0.001	1.380	1.183–1.609	< 0.001	1.482	1.274–1.724	<0.001
73–79 vs. 18–63	4.904	4.419–5.441	<0.001	3.258	2.763–3.843	<0.001	1.611	1.347–1.927	< 0.001	2.004	1.685–2.383	<0.001

Race			<0.001			<0.001						
Black vs. white	1.213	1.056–1.393	0.006	1.263	1.038–1.538	0.020	1.174	0.952–1.447	0.133	1.175	0.965–1.431	0.109
Others vs. white	0.683	0.579–0.805	< 0.001	0.703	0.558–0.885	0.003	0.766	0.606–0.968	0.025	0.739	0.587–0.931	0.010

Histology type												
ILC vs. IDC	–	–	0.627	–	–	0.351	–	–	0.576	–	–	0.527

Laterality												
Right vs. left	–	–	0.119	0.855	0.756–0.966	0.012	0.860	0.759–0.975	0.018	0.860	0.761–0.972	0.016

Grade			< 0.001			<0.001						
II vs. I	1.207	1.068–1.364	0.003	2.616	1.964–3.486	<0.001	2.651	1.993–3.526	<0.001	2.658	1.994–3.542	<0.001
III/IV vs. I	1.436	1.255–1.642	<0.001	3.435	2.567–4.596	<0.001	3.399	2.521–4.583	<0.001	3.426	2.549–4.604	<0.001

T stage			<0.001			<0.001						
T1b vs. T1a	1.081	0.866–1.350	0.490	0.834	0.555–1.254	0.384	0.838	0.561–1.251	0.386	0.842	0.560–1.266	0.408
T1c vs. T1a	1.463	1.194–1.793	<0.001	1.348	0.947–1.920	0.097	1.262	0.891–1.788	0.191	1.303	0.913–1.859	0.145
T2 vs. T1a	2.086	1.697–2.563	<0.001	2.398	1.690–3.403	<0.001	2.196	1.553–3.105	< 0.001	2.312	1.625–3.291	<0.001

N stage			<0.001			<0.001						
N1 vs. N0	1.455	1.322–1.602	<0.001	1.859	1.613–2.141	<0.001	1.901	1.636–2.209	<0.001	1.939	1.678–2.242	<0.001
N2 vs. N0	2.491	2.129–2.914	<0.001	3.847	3.141–4.712	<0.001	3.776	3.037–4.696	<0.001	3.969	3.231–4.875	<0.001
N3 vs. N0	4.567	3.763–5.541	<0.001	7.081	5.603–8.949	<0.001	6.353	4.897–8.242	<0.001	6.853	5.411–8.680	<0.001

ER												
Positive vs. negative	0.824	0.719–0.945	0.005	—	—	0.099	—	—	0.344	—	—	0.214

PR												
Positive vs. negative	0.851	0.763–0.949	0.004	0.625	0.550–0.711	<0.001	0.641	0.543–0.757	<0.001	0.644	0.549–0.755	<0.001

Treatment												
BCT vs. mastectomy	0.734	0.672–0.802	<0.001	0.660	0.576–0.755	<0.001	0.709	0.617–0.815	<0.001	0.686	0.598–0.786	<0.001

Chemotherapy												
Yes vs. no or unknown	0.765	0.695–0.843	<0.001	—	—	0.381	—	—	0.809	—	—	0.699

CLBC, centrally located breast cancer; HR, hazard ratio; CI, confidence interval; ILC, invasive lobular carcinoma; IDC, invasive ductal carcinoma; ER, estrogen receptor; PR, progesterone receptor; BCT, breast-conserving therapy.

**Table 7:** Multivariate survival analysis of prognostic factors among BCT patients with tumor located in the central portion and other quadrants.

	Cox-OS			Cox-BCSS			Fine–Gray			CS		
	HR	95% CI	*p*-value	HR	95% CI	*p*-value	HR	95% CI	*p*-value	HR	95% CI	*p*-value
UOBC vs. CLBC	0.932	0.869–0.999	0.047	—	—	0.319	—	—	0.578	—	—	0.482
UOBC vs. CLBC (matched)	0.851	0.773–0.938	0.001	—	—	0.136	—	—	0.410	—	—	0.290
UIBC vs. CLBC	—	—	0.715	1.210	1.071—1.366	0.002	1.221	1.079—1.381	0.002	1.214	1.075—1.371	0.002
LOBC vs. CLBC	—	—	0.992	1.161	1.022—1.319	0.022	1.170	1.02—1.333	0.018	1.162	1.023—1.321	0.021
LIBC vs. CLBC	1.135	1.04—1.233	0.003	1.360	1.194—1.550	<0.001	1.391	1.218—1.588	<0.001	1.383	1.213—1.576	<0.001

BCT, breast-conserving therapy; UOBC, upper-outer breast cancer; CLBC, centrally located breast cancer; UIBC, upper-inner breast cancer; LOBC, lower-outer breast cancer; LIBC.

## Data Availability

The datasets analyzed during the current study are available in the SEER database. https://seer.cancer.gov/.

## Supplementary Materials

Supplementary Materials Figure S1. Survival outcome in each subgroup among UIBC and CLBC patients who underwent BCT. (A) OS in the Cox proportional hazards model. (B) BCSS in the Cox proportional hazards model. (C) Fine–Gray model in the competing risks analysis. (D) CS model in the competing risks analysis. UIBC, upper-inner breast cancer; CLBC, centrally located breast cancer; BCT, breast-conserving therapy; OS, overall survival; BCSS, breast cancer-specific survival; CS, cause specific. Figure S2. Survival outcome in each subgroup among LOBC and CLBC patients who underwent BCT. (A) OS in the Cox proportional hazards model. (B) BCSS in the Cox proportional hazards model. (C) Fine-gray model in the competing risks analysis. (D) CS model in the competing risks analysis. LOBC, lower-outer breast cancer; CLBC, centrally located breast cancer; BCT, breast-conserving therapy; OS, overall survival; BCSS, breast cancer-specific survival; CS, cause specific. Figure S3. Survival outcome in each subgroup among LIBC and CLBC patients who underwent BCT. (A) OS in the Cox proportional hazards model. (B) BCSS in the Cox proportional hazards model. (C) Fine–Gray model in the competing risks analysis. (D) CS model in the competing risks analysis. LIBC, lower-inner breast cancer; CLBC, centrally located breast cancer; BCT, breast-conserving therapy; OS, overall survival; BCSS, breast cancer-specific survival; CS, cause-specific. Table S1. Multivariate survival analysis of prognostic factors among UOBC and CLBC patients who underwent BCT in the whole cohort. Table S2. Multivariate survival analysis of prognostic factors among UOBC and CLBC patients who underwent BCT in the matched cohort. Table S3. Multivariate survival analysis of prognostic factors among UIBC and CLBC patients who underwent BCT. Table S4. Multivariate survival analysis of prognostic factors among LOBC and CLBC patients who underwent BCT. Table S5. Multivariate survival analysis of prognostic factors among LIBC and CLBC patients who underwent BCT. Table S6. Multivariate survival analysis of prognostic factors among LIBC and CLBC patients with negative IMN who underwent BCT.
