# Supplementary material for: Survival Outcomes of Breast-Conserving Therapy versus Mastectomy in Early-Stage Breast Cancer, Including Centrally Located Breast Cancer: A SEER-Based Study
Source: Breast J. 2022 Aug 27;2022:5325556. doi: 10.1155/2022/5325556 (PMC9440848; doi:10.1155/2022/5325556)
Supplement: Supplementary Materials — Figure S1. Survival outcome in each subgroup among UIBC and CLBC patients who underwent BCT. (A) OS in the Cox proportional hazards model. (B) BCSS in the Cox proportional hazards model. (C) Fine–Gray model in the competing risks analysis. (D) CS model in the competing risks analysis. UIBC, upper-inner breast cancer; CLBC, centrally located breast cancer; BCT, breast-conserving therapy; OS, overall survival; BCSS, breast cancer-specific survival; CS, cause specific. Figure S2. Survival outcome in each subgroup among LOBC and CLBC patients who underwent BCT. (A) OS in the Cox proportional hazards model. (B) BCSS in the Cox proportional hazards model. (C) Fine-gray model in the competing risks analysis. (D) CS model in the competing risks analysis. LOBC, lower-outer breast cancer; CLBC, centrally located breast cancer; BCT, breast-conserving therapy; OS, overall survival; BCSS, breast cancer-specific survival; CS, cause specific. Figure S3. Survival outcome in each subgroup among LIBC and CLBC patients who underwent BCT. (A) OS in the Cox proportional hazards model. (B) BCSS in the Cox proportional hazards model. (C) Fine–Gray model in the competing risks analysis. (D) CS model in the competing risks analysis. LIBC, lower-inner breast cancer; CLBC, centrally located breast cancer; BCT, breast-conserving therapy; OS, overall survival; BCSS, breast cancer-specific survival; CS, cause-specific. Table S1. Multivariate survival analysis of prognostic factors among UOBC and CLBC patients who underwent BCT in the whole cohort. Table S2. Multivariate survival analysis of prognostic factors among UOBC and CLBC patients who underwent BCT in the matched cohort. Table S3. Multivariate survival analysis of prognostic factors among UIBC and CLBC patients who underwent BCT. Table S4. Multivariate survival analysis of prognostic factors among LOBC and CLBC patients who underwent BCT. Table S5. Multivariate survival analysis of prognostic factors among LIBC and CLBC patients who under [file 5325556.f1.zip › 5325556.f1/Table S1 (1).pdf]

Table S1. Multivariate survival analysis of prognostic factors among UOBC and CLBC patients who u

	Cox-OS			Cox-BCSS	
	HR	95% CI	p-value	HR	95% CI
Year of diagnosis			< 0.001		
2000-2003 vs. 2008-2015	1.422	1.345-1.503	< 0.001	1.944	1.797-2.103
2004-2007 vs. 2008-2015	1.185	1.121-1.252	< 0.001	1.295	1.195-1.403
Age, years			< 0.001		
64-72 vs. 18-63	2.450	2.328-2.579	< 0.001	1.580	1.459-1.712
73-79 vs. 18-63	5.421	5.138-5.719	< 0.001	3.174	2.895-3.479
Race			< 0.001		
Black vs. White	1.457	1.367-1.552	< 0.001	1.423	1.306-1.550
Others vs. White	0.787	0.719-0.862	< 0.001	0.856	0.755-0.971
Histology type					
ILC vs. IDC	-	-	0.273	1.183	1.040-1.346
Laterality					
Right vs. Left	-	-	0.079	0.935	0.879-0.993
Grade			< 0.001		
II vs. I	1.169	1.104-1.238	< 0.001	1.978	1.748-2.240
III/IV vs. I	1.439	1.347-1.537	< 0.001	2.769	2.433-3.152
T stage			< 0.001		
T1b vs. T1a	1.279	1.150-1.423	< 0.001	1.366	1.079-1.731
T1c vs. T1a	1.587	1.434-1.757	< 0.001	2.341	1.879-2.916
T2 vs. T1a	2.364	2.125-2.629	< 0.001	4.081	3.272-5.090
N stage			< 0.001		
N1 vs. N0	1.474	1.400-1.552	< 0.001	1.940	1.807-2.083
N2 vs. N0	2.672	2.454-2.909	< 0.001	3.785	3.423-4.184
N3 vs. N0	3.977	3.566-4.436	< 0.001	5.773	5.104-6.529
ER					
Positive vs. Negative	0.786	0.734-0.8843	< 0.001	0.740	0.673-0.813
PR					
Positive vs. Negative	0.838	0.790-0.888	< 0.001	0.694	0.635-0.758
Location					
UOBC vs. CLBC	0.932	0.869-0.999	0.047	-	-
Chemotherapy					
Yes vs. No or unknown	0.762	0.722-0.805	< 0.001	-	-

UOBC, upper-outer breast cancer; CLBC, centrally located breast cancer; BCT, breast-conserving t
PR, progesterone receptor.

underwent BCT in the whole cohort

	Fine-Gray			CS		
p-value	HR	95% CI	p-value	HR	95% CI	p-value
< 0.001						
< 0.001	1.668	1.543-1.803	< 0.001	1.618	1.496-1.750	< 0.001
< 0.001	1.200	1.108-1.300	< 0.001	1.183	1.093-1.280	< 0.001
< 0.001						
< 0.001	1.341	1.236-1.455	< 0.001	1.403	1.294-1.521	< 0.001
< 0.001	1.966	1.783-2.167	< 0.001	2.215	2.010-2.441	< 0.001
< 0.001						
< 0.001	1.386	1.267-1.516	< 0.001	1.409	1.293-1.535	< 0.001
0.016	0.874	0.770-0.993	0.038	0.870	0.767-0.987	0.030
0.011	1.231	1.086-1.395	0.001	1.215	1.068-1.382	0.003
0.029	0.939	0.882-0.999	0.047	0.938	0.883-0.997	0.039
< 0.001						
< 0.001	1.970	1.742-2.228	< 0.001	1.978	1.747-2.240	< 0.001
< 0.001	2.730	2.396-3.111	< 0.001	2.755	2.417-3.141	< 0.001
< 0.001						
0.010	1.292	1.020-1.637	0.034	1.314	1.037-1.665	0.024
< 0.001	2.178	1.746-2.717	< 0.001	2.243	1.798-2.798	< 0.001
< 0.001	3.804	3.041-4.758	< 0.001	3.944	3.154-4.932	< 0.001
< 0.001						
< 0.001	1.917	1.778-2.066	< 0.001	1.934	1.796-2.082	< 0.001
< 0.001	3.731	3.350-4.156	< 0.001	3.794	3.421-4.206	< 0.001
< 0.001	5.734	5.006-6.568	< 0.001	5.882	5.193-6.664	< 0.001
< 0.001	0.742	0.671-0.821	< 0.001	0.741	0.674-0.815	< 0.001
< 0.001	0.692	0.632-0.759	< 0.001	0.689	0.630-0.753	< 0.001
0.319	-	-	0.578	-	-	0.482
0.353	-	-	0.312	-	-	0.778

herapy; ILC, invasive lobular carcinoma; ILC, invasive lobular carcinoma; ER, estrogen re
