# Supplementary material for: Survival Outcomes of Breast-Conserving Therapy versus Mastectomy in Early-Stage Breast Cancer, Including Centrally Located Breast Cancer: A SEER-Based Study
Source: Breast J. 2022 Aug 27;2022:5325556. doi: 10.1155/2022/5325556 (PMC9440848; doi:10.1155/2022/5325556)
Supplement: Supplementary Materials — Figure S1. Survival outcome in each subgroup among UIBC and CLBC patients who underwent BCT. (A) OS in the Cox proportional hazards model. (B) BCSS in the Cox proportional hazards model. (C) Fine–Gray model in the competing risks analysis. (D) CS model in the competing risks analysis. UIBC, upper-inner breast cancer; CLBC, centrally located breast cancer; BCT, breast-conserving therapy; OS, overall survival; BCSS, breast cancer-specific survival; CS, cause specific. Figure S2. Survival outcome in each subgroup among LOBC and CLBC patients who underwent BCT. (A) OS in the Cox proportional hazards model. (B) BCSS in the Cox proportional hazards model. (C) Fine-gray model in the competing risks analysis. (D) CS model in the competing risks analysis. LOBC, lower-outer breast cancer; CLBC, centrally located breast cancer; BCT, breast-conserving therapy; OS, overall survival; BCSS, breast cancer-specific survival; CS, cause specific. Figure S3. Survival outcome in each subgroup among LIBC and CLBC patients who underwent BCT. (A) OS in the Cox proportional hazards model. (B) BCSS in the Cox proportional hazards model. (C) Fine–Gray model in the competing risks analysis. (D) CS model in the competing risks analysis. LIBC, lower-inner breast cancer; CLBC, centrally located breast cancer; BCT, breast-conserving therapy; OS, overall survival; BCSS, breast cancer-specific survival; CS, cause-specific. Table S1. Multivariate survival analysis of prognostic factors among UOBC and CLBC patients who underwent BCT in the whole cohort. Table S2. Multivariate survival analysis of prognostic factors among UOBC and CLBC patients who underwent BCT in the matched cohort. Table S3. Multivariate survival analysis of prognostic factors among UIBC and CLBC patients who underwent BCT. Table S4. Multivariate survival analysis of prognostic factors among LOBC and CLBC patients who underwent BCT. Table S5. Multivariate survival analysis of prognostic factors among LIBC and CLBC patients who under [file 5325556.f1.zip › 5325556.f1/Table S4 (1).pdf]

Table S4. Multivariate survival analysis of prognostic factors among LOBC and CLBC patients who u

	Cox-OS			Cox-BCSS	
	HR	95% CI	p-value	HR	95% CI
Year of diagnosis			< 0.001		
2000-2003 vs. 2008-2015	1.382	1.243-1.537	< 0.001	1.952	1.679-2.269
2004-2007 vs. 2008-2015	1.164	1.048-1.291	0.004	1.393	1.200-1.617
Age, years			< 0.001		
64-72 vs. 18-63	2.256	2.046-2.489	< 0.001	1.336	1.151-1.552
73-79 vs. 18-63	4.957	4.478-5.487	< 0.001	2.490	2.091-2.964
Race			< 0.001		
Black vs. White	1.291	1.136-1.467	< 0.001	1.216	1.023-1.446
Others vs. White	0.757	0.637-0.898	0.001	0.810	0.642-1.023
Histology type					
ILC vs. IDC	-	-	0.547	-	-
Laterality					
Right vs. Left	-	-	0.937	-	-
Grade			< 0.001		
II vs. I	1.203	1.076-1.344	0.001	2.181	1.719-2.769
III/IV vs. I	1.528	1.349-1.731	< 0.001	3.202	2.511-4.084
T stage			< 0.001		
T1b vs. T1a	1.335	1.086-1.641	0.006	1.172	0.791-1.737
T1c vs. T1a	1.621	1.331-1.975	< 0.001	1.820	1.268-2.612
T2 vs. T1a	2.518	2.050-3.094	< 0.001	3.508	2.437-5.049
N stage			< 0.001		
N1 vs. N0	1.481	1.344-1.631	< 0.001	1.803	1.578-2.060
N2 vs. N0	2.682	2.279-3.155	< 0.001	3.630	2.990-4.406
N3 vs. N0	4.740	3.867-5.809	< 0.001	6.460	5.133-8.131
ER					
Positive vs. Negative	0.816	0.731-0.911	< 0.001	-	-
PR					
Positive vs. Negative	-	-	0.348	0.706	0.623-0.800
Location					
LOBC vs. CLBC	-	-	0.992	1.161	1.022-1.319
Chemotherapy					
Yes vs. No or unknown	0.811	0.732-0.899	< 0.001	-	-

LOBC, lower-outer breast cancer; CLBC, centrally located breast cancer; BCT, breast-conserving th
PR, progesterone receptor.

underwent BCT

	Fine-Gray			CS		
p-value	HR		p-value	HR		p-value
< 0.001						
< 0.001	1.691	1.457-1.963	< 0.001	1.622	1.396-1.884	< 0.001
< 0.001	1.303	1.122-1.514	< 0.001	1.273	1.097-1.478	0.002
< 0.001						
< 0.001	1.174	1.007-1.368	0.040	1.227	1.054-1.429	0.008
< 0.001	1.590	1.319-1.916	< 0.001	1.826	1.517-2.198	< 0.001
0.011						
0.027	1.197	1.002-1.431	0.048	1.205	1.013-1.432	0.035
0.076	0.850	0.672-1.075	0.175	0.841	0.666-1.061	0.144
0.310	-	-	0.075	-	-	0.100
0.432	-	-	0.391	-	-	0.414
< 0.001						
< 0.001	2.116	1.666-2.689	< 0.001	2.132	1.678-2.709	< 0.001
< 0.001	3.109	2.410-4.010	< 0.001	3.125	2.436-4.009	< 0.001
< 0.001						
0.429	1.094	0.739-1.619	0.653	1.114	0.751-1.651	0.591
0.001	1.608	1.116-2.315	0.011	1.654	1.148-2.384	0.007
< 0.001	2.986	2.061-4.326	< 0.001	3.108	2.147-4.499	< 0.001
< 0.001						
< 0.001	1.735	1.504-2.001	< 0.001	1.758	1.530-2.020	< 0.001
< 0.001	3.317	2.692-4.085	< 0.001	3.393	2.777-4.144	< 0.001
< 0.001	5.513	4.273-7.111	< 0.001	5.752	4.545-7.278	< 0.001
0.060	-	-	0.193	-	-	0.133
< 0.001	0.760	0.639-0.904	0.002	0.764	0.649-0.899	0.001
0.022	1.170	1.028-1.333	0.018	1.162	1.023-1.321	0.021
0.075	1.207	1.022-1.425	0.026	1.176	1.003-1.379	0.046

erapy; ILC, invasive lobular carcinoma; ILC, invasive lobular carcinoma; ER, estrogen rec
