# Supplementary figures and images for: Survival Outcomes of Breast-Conserving Therapy versus Mastectomy in Early-Stage Breast Cancer, Including Centrally Located Breast Cancer: A SEER-Based Study
Source: Breast J. 2022 Aug 27;2022:5325556. doi: 10.1155/2022/5325556 (PMC9440848; doi:10.1155/2022/5325556)

Figure S1

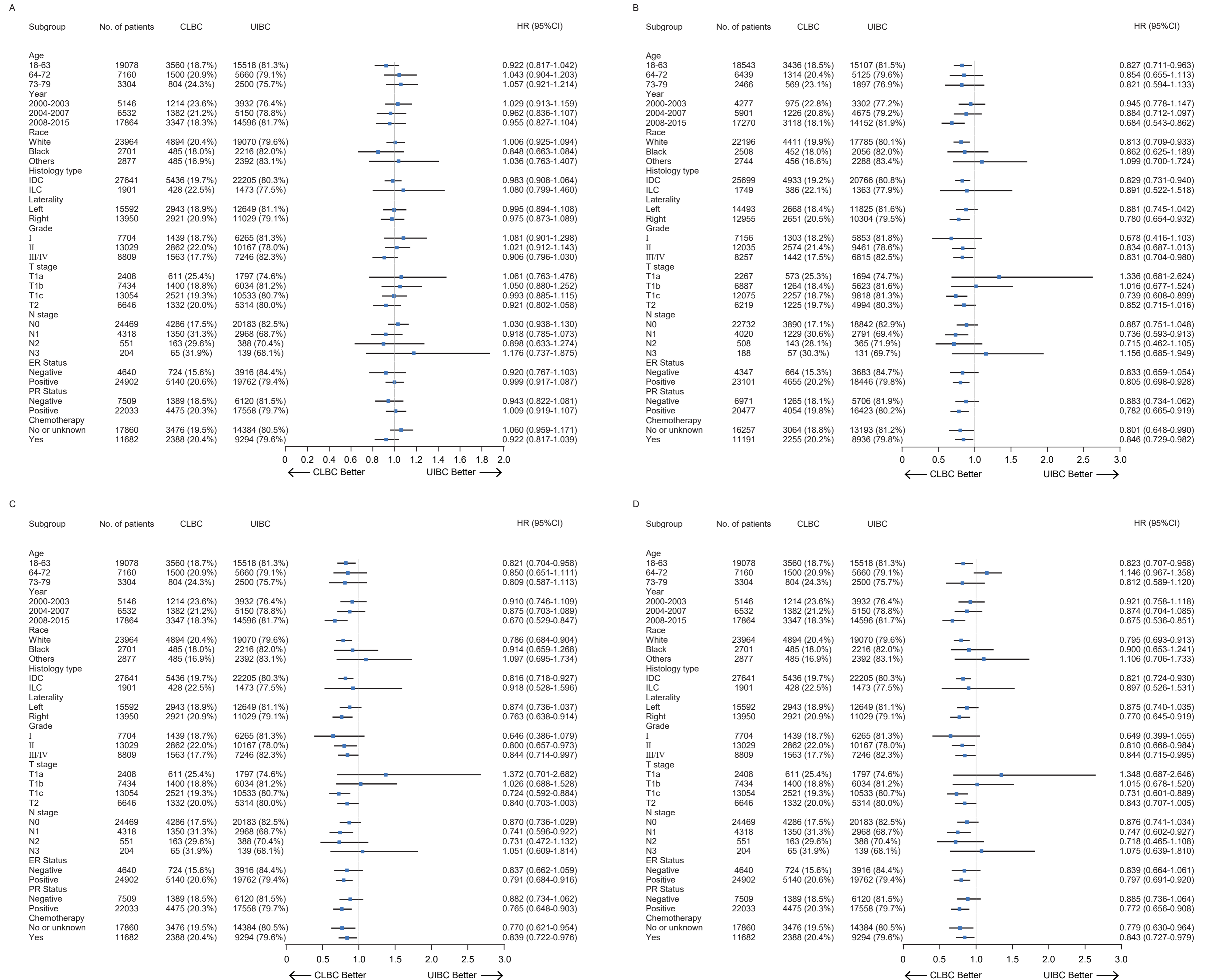

Supplement: Supplementary Materials — Figure S1. Survival outcome in each subgroup among UIBC and CLBC patients who underwent BCT. (A) OS in the Cox proportional hazards model. (B) BCSS in the Cox proportional hazards model. (C) Fine–Gray model in the competing risks analysis. (D) CS model in the competing risks analysis. UIBC, upper-inner breast cancer; CLBC, centrally located breast cancer; BCT, breast-conserving therapy; OS, overall survival; BCSS, breast cancer-specific survival; CS, cause specific. Figure S2. Survival outcome in each subgroup among LOBC and CLBC patients who underwent BCT. (A) OS in the Cox proportional hazards model. (B) BCSS in the Cox proportional hazards model. (C) Fine-gray model in the competing risks analysis. (D) CS model in the competing risks analysis. LOBC, lower-outer breast cancer; CLBC, centrally located breast cancer; BCT, breast-conserving therapy; OS, overall survival; BCSS, breast cancer-specific survival; CS, cause specific. Figure S3. Survival outcome in each subgroup among LIBC and CLBC patients who underwent BCT. (A) OS in the Cox proportional hazards model. (B) BCSS in the Cox proportional hazards model. (C) Fine–Gray model in the competing risks analysis. (D) CS model in the competing risks analysis. LIBC, lower-inner breast cancer; CLBC, centrally located breast cancer; BCT, breast-conserving therapy; OS, overall survival; BCSS, breast cancer-specific survival; CS, cause-specific. Table S1. Multivariate survival analysis of prognostic factors among UOBC and CLBC patients who underwent BCT in the whole cohort. Table S2. Multivariate survival analysis of prognostic factors among UOBC and CLBC patients who underwent BCT in the matched cohort. Table S3. Multivariate survival analysis of prognostic factors among UIBC and CLBC patients who underwent BCT. Table S4. Multivariate survival analysis of prognostic factors among LOBC and CLBC patients who underwent BCT. Table S5. Multivariate survival analysis of prognostic factors among LIBC and CLBC patients who under [file 5325556.f1.zip › 5325556.f1/Figure S1.pdf]

Figure S2

A

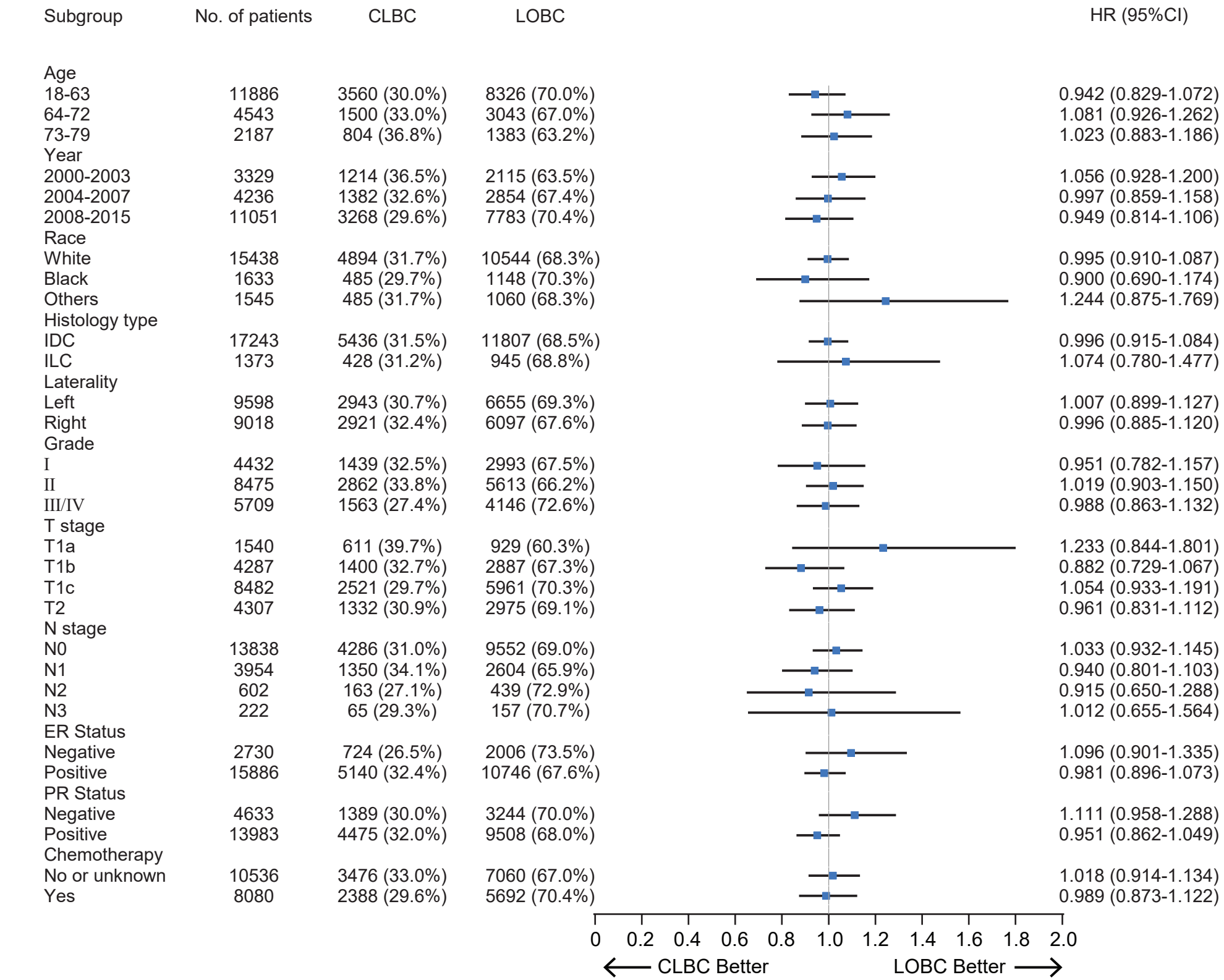

B

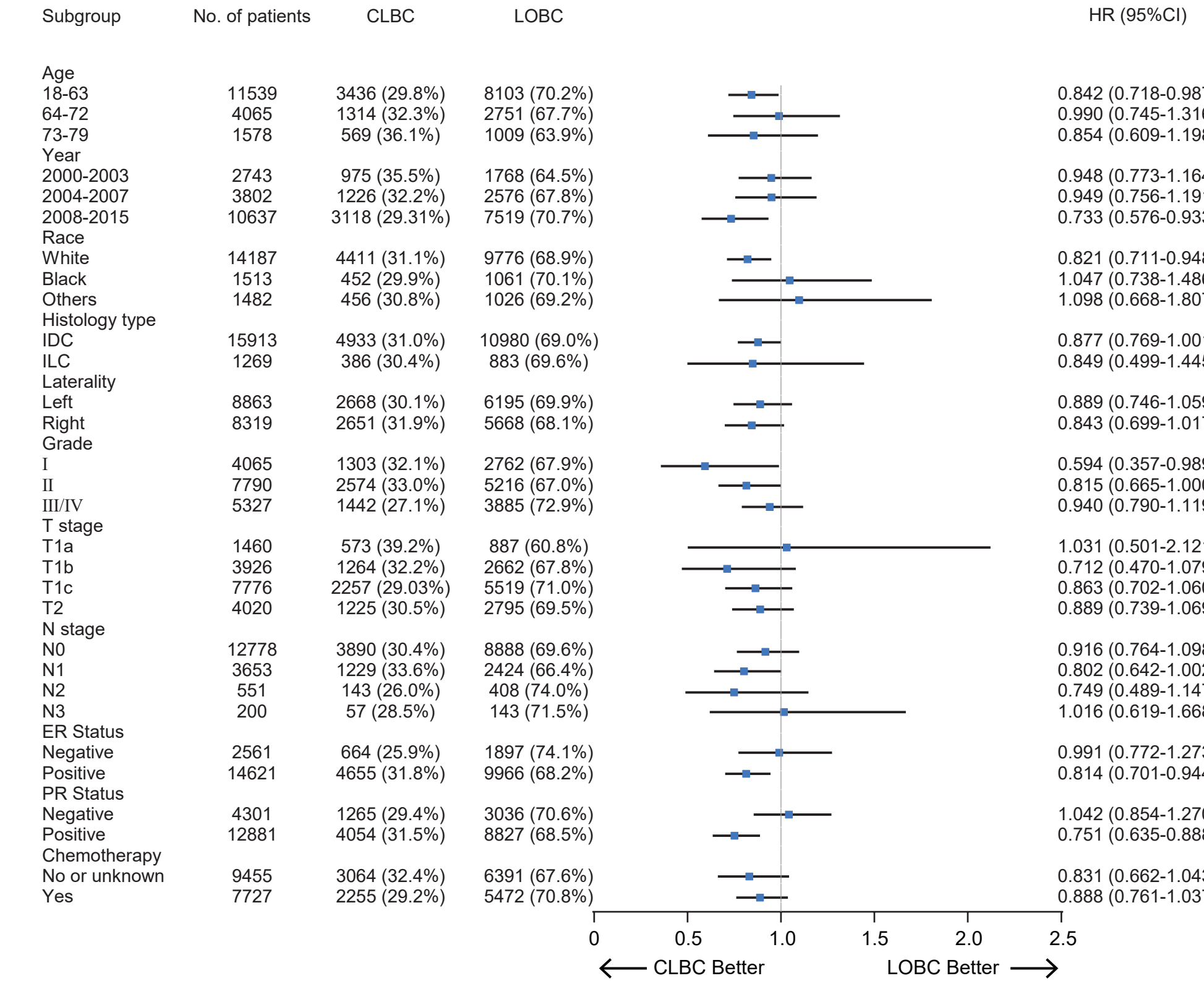

C

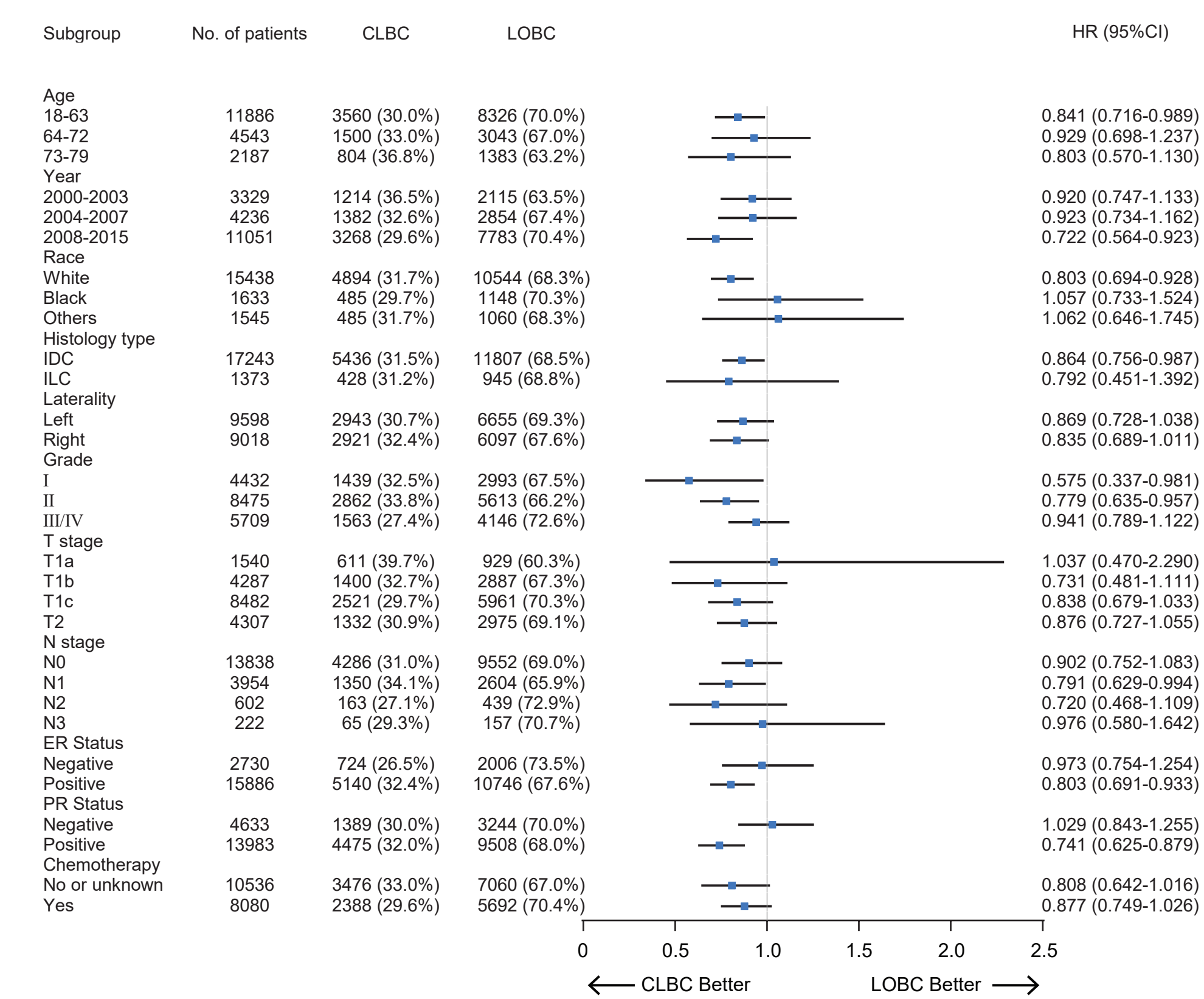

D

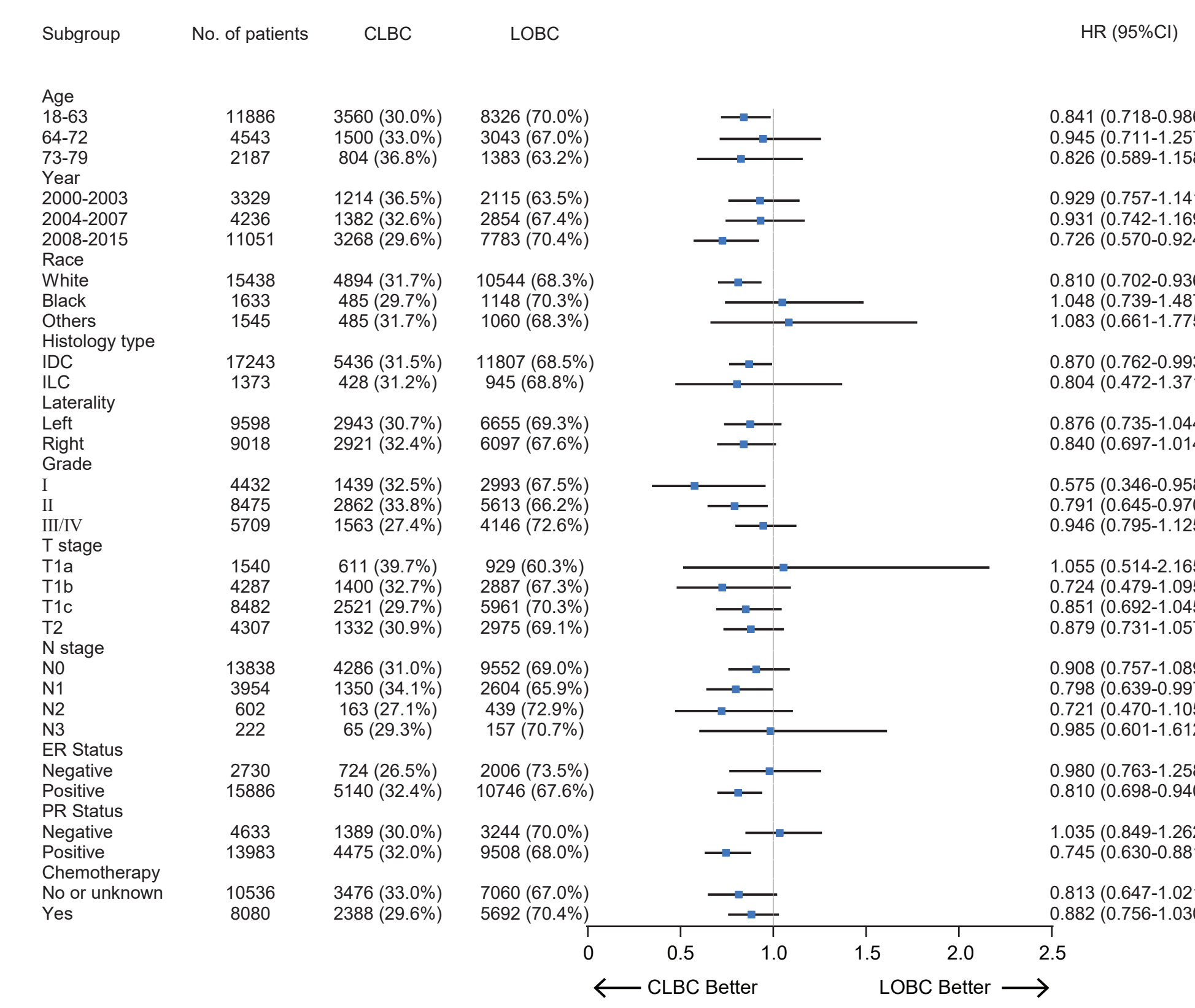

Supplement: Supplementary Materials — Figure S1. Survival outcome in each subgroup among UIBC and CLBC patients who underwent BCT. (A) OS in the Cox proportional hazards model. (B) BCSS in the Cox proportional hazards model. (C) Fine–Gray model in the competing risks analysis. (D) CS model in the competing risks analysis. UIBC, upper-inner breast cancer; CLBC, centrally located breast cancer; BCT, breast-conserving therapy; OS, overall survival; BCSS, breast cancer-specific survival; CS, cause specific. Figure S2. Survival outcome in each subgroup among LOBC and CLBC patients who underwent BCT. (A) OS in the Cox proportional hazards model. (B) BCSS in the Cox proportional hazards model. (C) Fine-gray model in the competing risks analysis. (D) CS model in the competing risks analysis. LOBC, lower-outer breast cancer; CLBC, centrally located breast cancer; BCT, breast-conserving therapy; OS, overall survival; BCSS, breast cancer-specific survival; CS, cause specific. Figure S3. Survival outcome in each subgroup among LIBC and CLBC patients who underwent BCT. (A) OS in the Cox proportional hazards model. (B) BCSS in the Cox proportional hazards model. (C) Fine–Gray model in the competing risks analysis. (D) CS model in the competing risks analysis. LIBC, lower-inner breast cancer; CLBC, centrally located breast cancer; BCT, breast-conserving therapy; OS, overall survival; BCSS, breast cancer-specific survival; CS, cause-specific. Table S1. Multivariate survival analysis of prognostic factors among UOBC and CLBC patients who underwent BCT in the whole cohort. Table S2. Multivariate survival analysis of prognostic factors among UOBC and CLBC patients who underwent BCT in the matched cohort. Table S3. Multivariate survival analysis of prognostic factors among UIBC and CLBC patients who underwent BCT. Table S4. Multivariate survival analysis of prognostic factors among LOBC and CLBC patients who underwent BCT. Table S5. Multivariate survival analysis of prognostic factors among LIBC and CLBC patients who under [file 5325556.f1.zip › 5325556.f1/Figure S2.pdf]

Figure S3

A

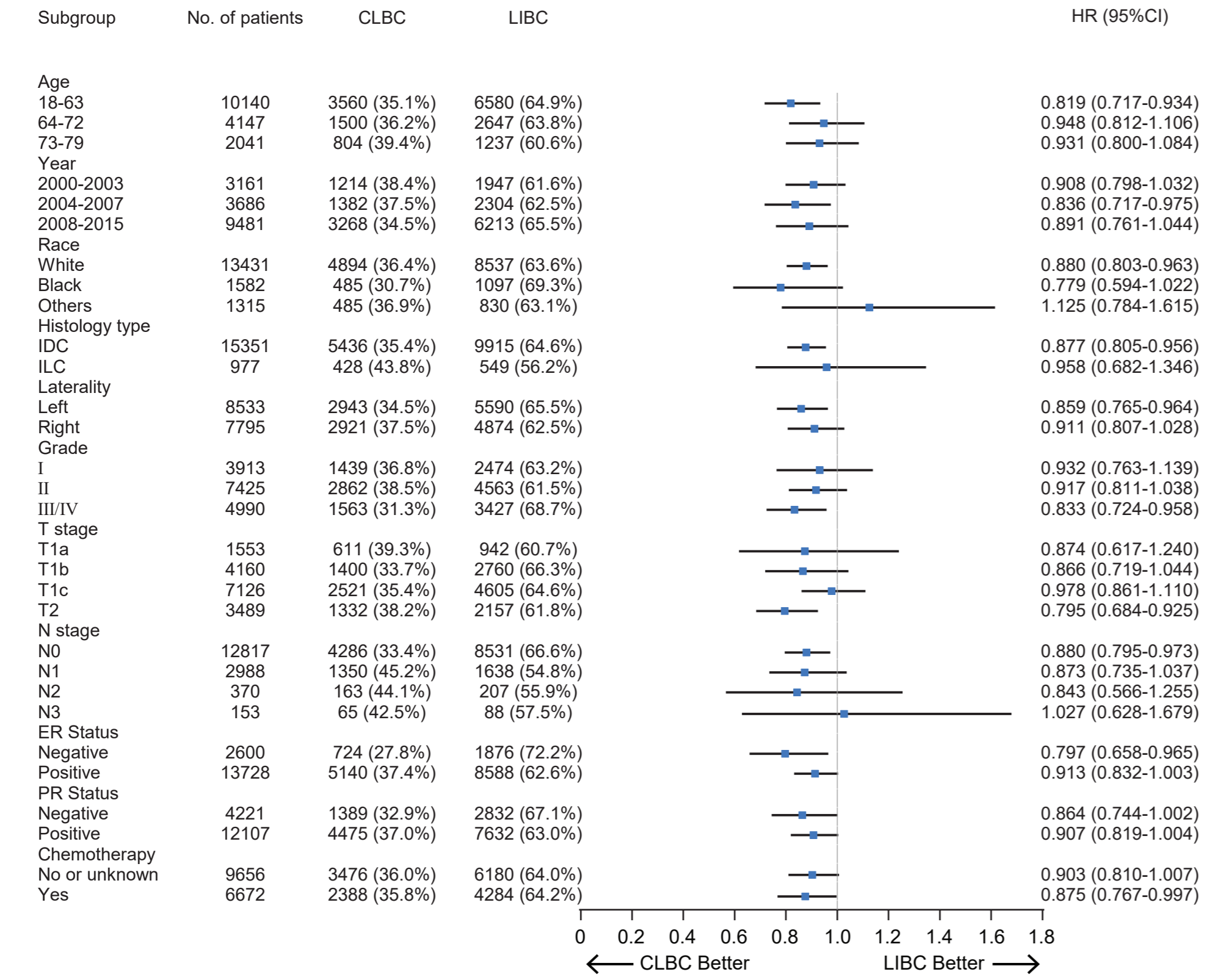

B

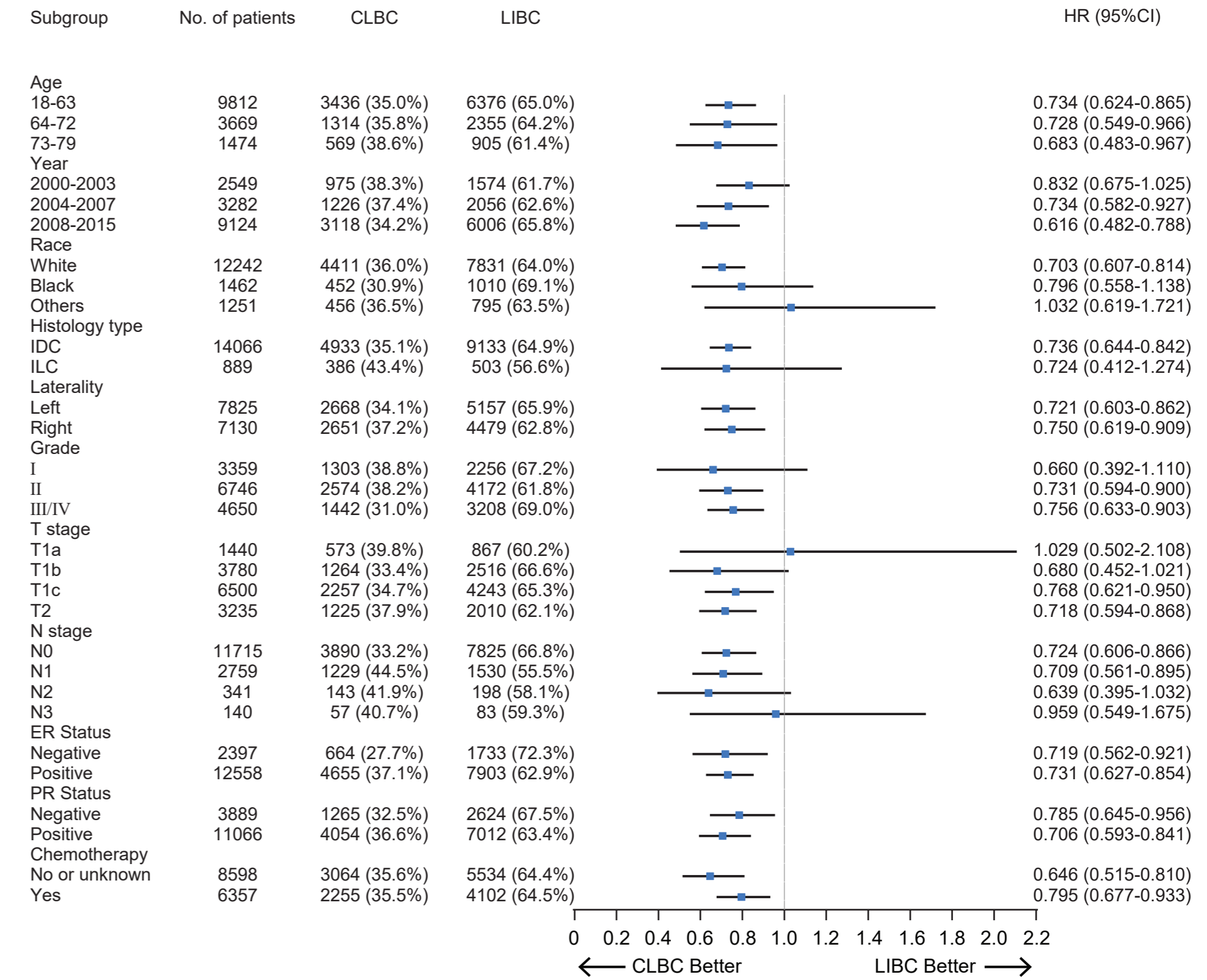

C

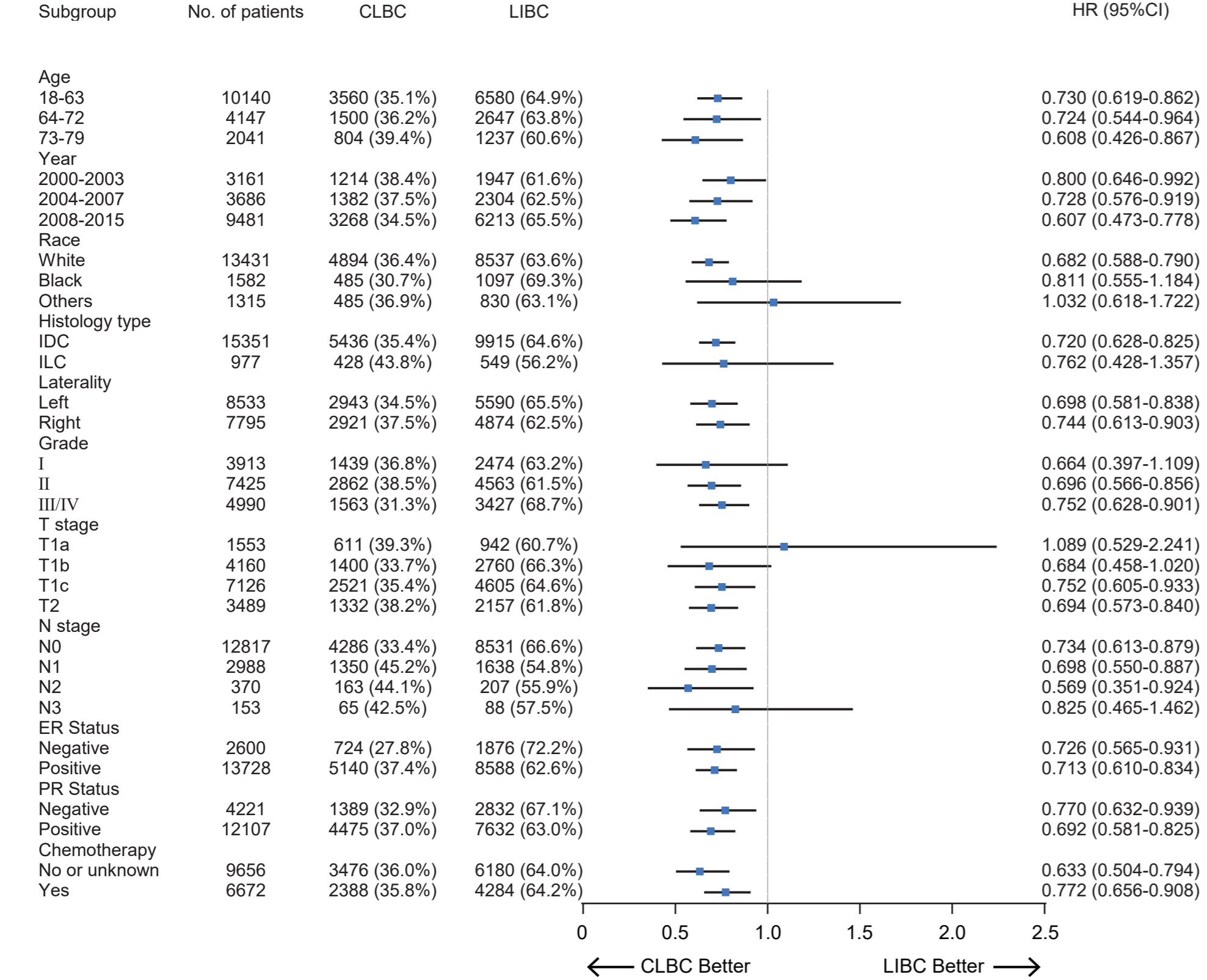

D

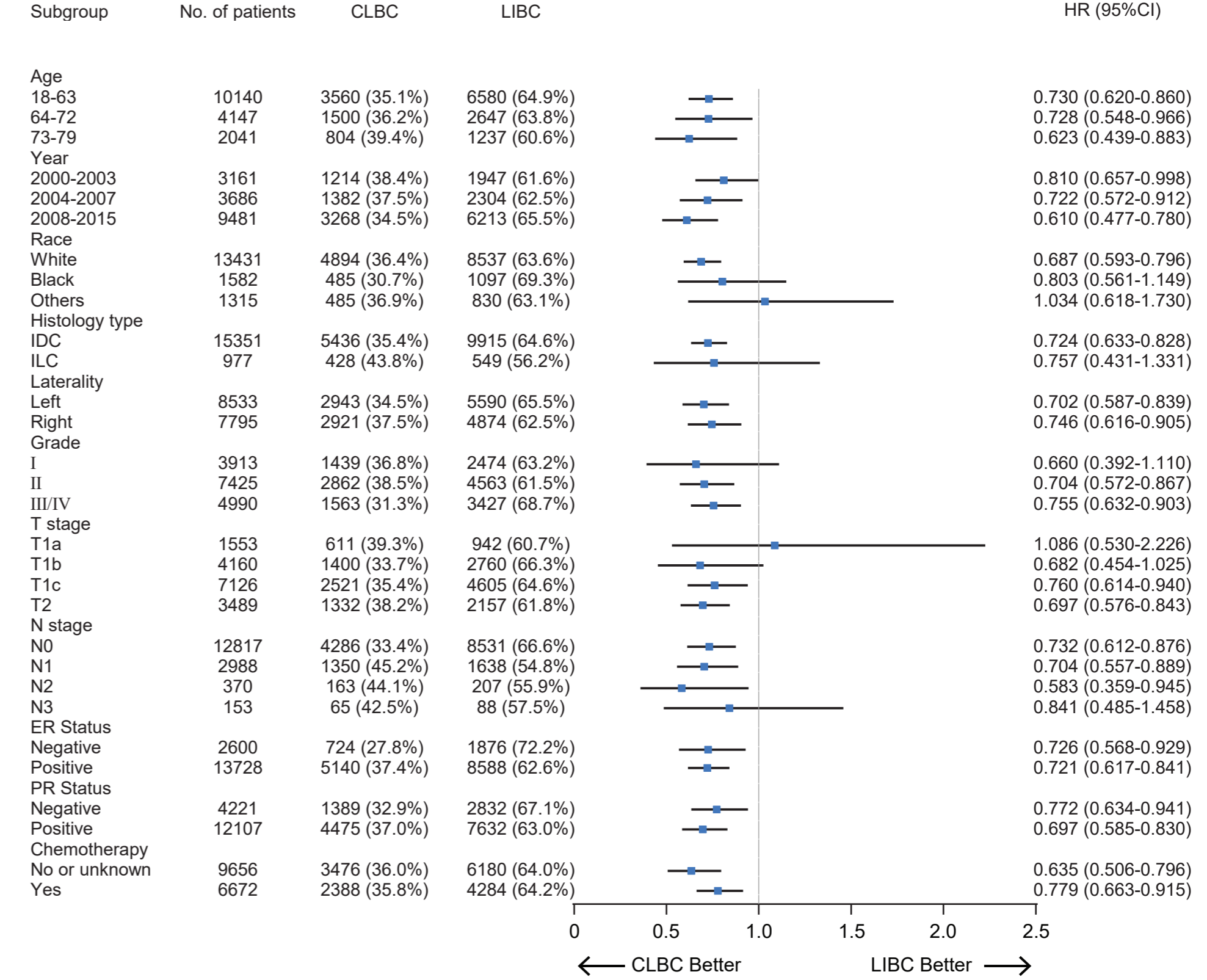

Supplement: Supplementary Materials — Figure S1. Survival outcome in each subgroup among UIBC and CLBC patients who underwent BCT. (A) OS in the Cox proportional hazards model. (B) BCSS in the Cox proportional hazards model. (C) Fine–Gray model in the competing risks analysis. (D) CS model in the competing risks analysis. UIBC, upper-inner breast cancer; CLBC, centrally located breast cancer; BCT, breast-conserving therapy; OS, overall survival; BCSS, breast cancer-specific survival; CS, cause specific. Figure S2. Survival outcome in each subgroup among LOBC and CLBC patients who underwent BCT. (A) OS in the Cox proportional hazards model. (B) BCSS in the Cox proportional hazards model. (C) Fine-gray model in the competing risks analysis. (D) CS model in the competing risks analysis. LOBC, lower-outer breast cancer; CLBC, centrally located breast cancer; BCT, breast-conserving therapy; OS, overall survival; BCSS, breast cancer-specific survival; CS, cause specific. Figure S3. Survival outcome in each subgroup among LIBC and CLBC patients who underwent BCT. (A) OS in the Cox proportional hazards model. (B) BCSS in the Cox proportional hazards model. (C) Fine–Gray model in the competing risks analysis. (D) CS model in the competing risks analysis. LIBC, lower-inner breast cancer; CLBC, centrally located breast cancer; BCT, breast-conserving therapy; OS, overall survival; BCSS, breast cancer-specific survival; CS, cause-specific. Table S1. Multivariate survival analysis of prognostic factors among UOBC and CLBC patients who underwent BCT in the whole cohort. Table S2. Multivariate survival analysis of prognostic factors among UOBC and CLBC patients who underwent BCT in the matched cohort. Table S3. Multivariate survival analysis of prognostic factors among UIBC and CLBC patients who underwent BCT. Table S4. Multivariate survival analysis of prognostic factors among LOBC and CLBC patients who underwent BCT. Table S5. Multivariate survival analysis of prognostic factors among LIBC and CLBC patients who under [file 5325556.f1.zip › 5325556.f1/Figure S3.pdf]
